# The amino acid composition of commercially available vegan meat and dairy analogues

**DOI:** 10.1017/S000711452510408X

**Published:** 2025-09-14

**Authors:** Jacintha Domić, Luc J. C. van Loon, Els Siebelink, Karin J. Borgonjen-van den Berg, Lisette C. P. G. M. de Groot, Pol Grootswagers

**Affiliations:** 1 Division of Human Nutrition and Health, Wageningen University, Wageningen, The Netherlands; 2 Department of Human Biology, Institute of Nutrition and Translational Research in Metabolism (NUTRIM), Maastricht University Medical Centre+, Maastricht, The Netherlands

**Keywords:** Protein, Plant based, Sustainable food, Meat substitute, Dairy substitute

## Abstract

Plant-based meat and dairy analogues contain less protein than their animal-based counterparts and rely on various plant protein sources, which frequently display incomplete amino acid (AA) profiles that do not reflect dietary requirements due to low quantities of one or more essential AA (EAA). There is little insight in the AA profiles of most of these plant-based analogues. We assessed the AA composition of forty plant-based meat and dairy analogues that were commercially available in The Netherlands in March 2023 and compared their EAA profile to dietary requirements and to the EAA profile of their meat and dairy counterparts. Total protein contents were lower in most analogues when compared with their animal-based counterparts (meat analogues, *n* 16 (80 %); lunch meats and cheese analogues, *n* 10 (100 %); milk and yoghurt analogues, *n* 9 (90 %)) and accompanied by lower EAA contents. In reference to dietary requirements, the sum of the total EAA contents was adequate in all but one of the analogues. Nevertheless, all analogues displayed deficiencies in one or more specific EAA. Methionine contents were most frequently low (*n* 39; 98 %), followed by lysine contents (*n* 11; 28 %). Essential AA compositions varied between analogues irrespective of the protein source(s) used. In conclusion, plant-based meat and dairy analogues exhibit incomplete EAA profiles, which may compromise adequate protein nutrition in plant-centred diets.

The growing demand for products free of animal-based ingredients has led to an increase in the production and consumption of plant-based meat and dairy analogues in high-income countries^(1,2)^. These analogues are developed to provide a more sustainable, plant-based substitution for meat and dairy based products, while mimicking their taste and texture. Nevertheless, plant-based meat and dairy analogues generally do not mimic the nutrient composition of their animal-based counterparts, as they generally contain less dietary protein and, as such, more carbohydrates, fat and/or dietary fiber^(3,4)^.

Dietary protein is an essential macronutrient for the growth and maintenance of body proteins, e.g. skeletal muscle protein^(5)^. The dietary protein provided by meat and dairy exhibits excellent quality due to their high digestibility and favorable amino acid (AA) profiles, while these factors are more variable in plant-based foods^(6–8)^. Amino acids are the main constituents of dietary protein. Nine of these dietary AA cannot be synthesised by the body, and are thus considered essential and must be obtained via food intake. Daily dietary requirements have been established for the essential AA (EAA; i.e. histidine, leucine, isoleucine, lysine, methionine, threonine, tryptophan, phenylalanine and valine). Additional requirements have been established for two AA that are considered conditionally essential: cysteine and tyrosine, as they can become essential under specific conditions^(5,9)^. The EAA profile of a protein source is considered complete when it provides all EAA, including cysteine and tyrosine, in quantities that are in line with human requirements. A more complete EAA profile increases the quality score and likely improves the functionality of the consumed protein. It is suggested that the consumption of a meal, or diet, that exhibits low quantities of one or more EAA when compared with human requirements leads to lower synthesis rates of bodily proteins, and successive consumption of such meals may over time lead to losses in muscle mass and bone density^(10–12)^.

Plant-based meat and dairy analogues are developed with proteins derived from various plant-based sources, e.g. legumes, nuts, seeds, grains, tubers, algae, fungi or combinations thereof. Proteins derived from these sources all exhibit varying AA profiles. While some protein sources have an EAA profile that aligns well with dietary requirements (e.g. potato protein), other protein sources (e.g. wheat, pea and oat protein) are more unbalanced and exhibit deficiencies in one or more specific amino acids^(11,13)^. These EAA profiles will not necessarily be reflected in the meat and dairy analogues that incorporate these respective sources, as these sources are incorporated in various forms (e.g. flours (∼50 % protein), protein concentrate (∼70 % protein) or protein isolate (∼90 % protein)), quantities and combinations. Furthermore, in the product development of meat and dairy analogues, several processing steps are used, such as heat treatment, extrusion and fermentation, which may further alter amino acid profiles and/or post-prandial bioavailability^(14–16)^. The AA profiles of most meat and dairy analogues available on the Dutch market are generally not presented or available to the consumers.

As meat and dairy analogues are becoming a growing part of the diet in high-income countries^(1,2)^, it is important to determine the AA composition of these products and to understand the extent to which they can indeed replace the dietary EAA that would otherwise be consumed via meat and dairy. This is particularly important for fully plant-based, i.e. vegan, analogues, as these contain no animal-derived protein. Furthermore, it is important to assess and report the amino acid profiles separately for each analogue to allow for these values to be used in nutrient composition databases and to allow for comparisons between products. In this study, we analysed and reported the amino acid profiles of forty unprepared meat and dairy analogues that are available on the Dutch market and compared these to their animal-based counterparts and the FAO reference amino acid pattern^(9)^. In addition, we secondarily assessed potential changes in the amino acid profile of one of the meat analogues following heat treatment.

## Methods

### Product selection

**Figure 1.**
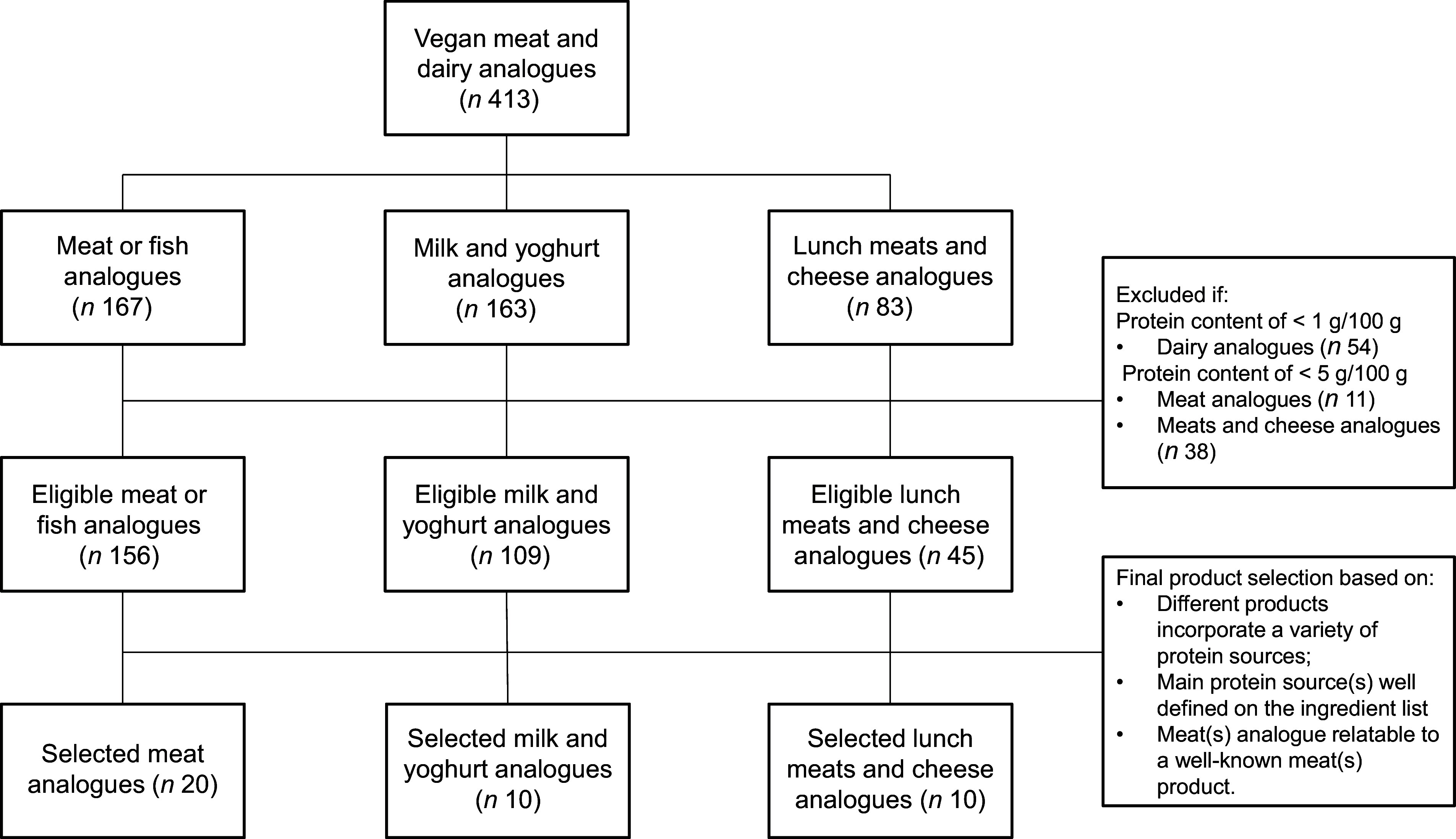
Flow chart regarding the selection procedure of the analysed meat and dairy analogues.

Analogues were selected in October 2022 from the online stores of the two largest supermarket chains in The Netherlands: Albert Heijn and Jumbo. The selection procedure is presented in Figure 1. In total, 167 meat analogues, 163 milk or yoghurt analogues and eighty-three plant-based lunch meats and cheese analogues (bread toppings) were available online at the respective supermarkets at that time. Milk and yoghurt analogues were considered eligible for analysis when the product contained more than 1 g protein per 100 g product. The other analogues were considered eligible for analysis when the products contained more than 5 g protein per 100 g product. Based on this criterium, 310 products were considered eligible for analysis. Subsequently, a final selection of twenty meat analogues, ten lunch meats- and cheese analogues (bread toppings) and ten milk and yoghurt analogues were made based on the following considerations: (1) the meat and dairy analogues that were to be analysed were based on varying protein sources and blends; (2) the main protein source(s) of the analogues were well defined on the ingredient list and (3) the meat(s) analogues were relatable to well-known meat products.

Furthermore, to allow for comparison between the analogues and their respective animal-based counterparts, three animal-based products were selected to be analysed as well. Beef is well known for its high protein quality, fast digestion rate and ability to provoke a net positive post-prandial protein balance^(6,17,18)^. We selected minced beef (98 % beef, 16 % fat) as a reference for all meat and lunch meats analogues. Bovine, semi-skimmed milk (1·5 % fat) and bovine Gouda cheese (31 % fat) were selected as the reference for milk and cheese products^(19)^. Finally, bovine yoghurt, bovine chocolate milk and bovine quark were selected as a reference for the chocolate milk, yoghurt and quark analogues, because these products exhibit different quantities of protein than bovine, semi-skimmed milk^(20)^. Assuming that the relative AA profile per g protein is similar, we calculated the EAA composition per portion of bovine semi-skimmed yoghurt, bovine full-fat chocolate milk and bovine semi-skimmed quark from the EAA composition of bovine, semi-skimmed milk.

### Product sampling

The analogues were purchased in March 2023 from different stores of the respective supermarkets located in Utrecht, Tilburg and Wageningen in The Netherlands. At that time, eleven of the initially selected products were not commercially available anymore and were replaced by other analogues that preferably, but not necessarily, were based on similar protein sources as the initially selected products. The animal-based comparatives were purchased in July 2024 from the same supermarkets located in Wageningen. If available, two packages of each product were purchased, with either package containing a different expiration date. In this way, we ensured that packages from different production batches were purchased and homogenised together. For three selected products (Vly unsweetened, Vly; Oatgurt Blueberry, Oatly; Plant-based Alternative for Quark, Albert Heijn), only packages with the same expiration date were available at the respective supermarkets and, as such, their homogenised samples only contained products from the same production batch.

An overview of the purchased products can be found in Table 1. The most frequently used sources of protein in the included meat analogues were soya, pea and wheat protein, which all were incorporated in 9 (45 %) different meat analogues. These protein sources were incorporated as the main source (i.e. first mentioned on the ingredient list), in 5 (25 %), 4 (20 %) and 4 (20 %) of these meat analogues, respectively. Regarding the selected lunch meats analogues, soya was incorporated in most (*n* 4 (50 %)) analogues as the main protein source. Oat and pea were the most frequently present protein sources in the selected products that fell within the milk and yoghurt analogue category (both *n* 4 (40 %)) and were the main protein source in 4 (40 %) and 2 (20 %) of these analogues, respectively. The two cheese analogues were based on almond and soya.

**Table 1.** Overview of the vegan meat and dairy analogues and animal-based counterparts

Product	Brand	Protein (g/100 g)^*^	Main plant-based protein sources
Meat analogues			
Chilli burger	BOON	8·6	Broad bean, brown bean
Chilli nuggets	BOON	8·6	Broad bean, brown bean, wheat, quinoa
Falafel burger	Albert Heijn	6·6	Chickpea
Plant-based burger 1	Albert Heijn	16·0	Field bean, pea
Plant-based minced meat 1	Albert Heijn	17·0	Field bean, pea
Weedburger umami master	The Dutch weed	10·4	Field bean, wheat, pea, seaweed
Vegetable burger	Vivera	11·4	Mixed vegetables, soya, potato
Plant-based sausage 1	Beyond meat	17·0	Pea
Plant-based burger 2	Beyond meat	17·0	Pea, rice
Plant-based minced meat 2	Beyond meat	15·0	Pea, rice
Plant-based Swedish balls	Albert Heijn	9·6	Pea, wheat
Plant-based chicken pieces 1	Vegetarian Butcher	21·0	Soya
Plant-based minced meat 3	Vegetarian Butcher	24·0	Soya
Sensational burger	Garden gourmet	14·0	Soya
Plant-based chicken pieces 2	Vivera	15·0	Soya, wheat
Plant-based chicken tenders	Vivera	15·0	Soya, wheat
Seitan sausage	Smaakt	27·0	Wheat
Plant-based soup balls	Albert Heijn	8·5	Wheat, pea, soya, potato
Plant-based minced meat 4	Jumbo	15·2	Wheat, soya, mushrooms
Plant-based chicken burger	Like meat	13·0	Wheat, soya, pea
Meats and cheese analogues			
Meats			
Plant-based luncheon meat 1	Quorn	7·1	Mycoprotein
Plant-based bacon 1	Garden gourmet	5·5	Pea
Plant-based grilled sausage	Albert heijn	8·3	Rice
Plant-based bacon 2	La Vie	14·9	Soya
Plant-based chicken filet	Jumbo	8·3	Soya
Plant-based luncheon meat 2	Vegetarian butcher	8·2	Soya
Plant-based sausage 2	Kips	7·1	Soya
Plant-based bacon 3	Albert heijn	19·0	Wheat
* *Cheese			
Mozzarella alternative	Mondarella	5·0	Almond
Melt me smoky	Wildwestland	8·3	Soya
Milk and yoghurt analogues			
Plant-based quark alternative unsweetened	Albert Heijn	2·7	Almond
Plant-based chocolate milk	Chocomel	1·8	Cashew nut, pea
Coco original	Abott Kinney’s	1·1	Coconut
Oat drink	Nature	1·6	Oat
Oat growth drink	Alpro	1·8	Oat, pea
Oatgurt blueberry	Oatly	1·3	Oat, potato
Oatgurt Greek style	Abbot Kinney’s	3·3	Oat, potato
Plant-based not milk	Wunda	2·2	Pea
Vly unsweetened	Vly	2·5	Pea
Plant-based quark alternative soya	Albert Heijn	5·7	Soya
Animal based counterparts			
Minced beef	Albert Heijn	20	NA
Bovine, semi-skimmed milk	Albert Heijn	3·6	NA
Bovine gouda cheese	Albert Heijn	24	NA
Bovine semi-skimmed yoghurt	Albert Heijn	4·2	NA
Bovine full-fat chocolate milk	Albert Heijn	3·3	NA
Bovine semi-skimmed quark	Albert Heijn	7·5	NA

* Protein is presented as g/100 g product as specified on the front label of the packaging.

### Sample preparation

All products were homogenised in raw form prior to analysis of AA content. If necessary, water was added to the products to facilitate homogenisation, and this was subsequently accounted for during data analysis. The homogenised samples were stored in at −20°C until further analysis. Additionally, one meat analogue (Sensational Burger, Garden Gourmet) was homogenised following two different preparation methods, as specified on the packaging: (1) following preparation in the oven at 200°C for 11 min; (2) following preparation in a frying pan, by baking the burgers for 3 min on each side on medium temperature using 51 g of sunflower oil following 60 s of preheating the pan and sunflower oil. The frying pan was weighed before preparation of the burger, and subsequently after preparation including the residual sunflower oil to the nearest 0·1 kg with a calibrated digital scale (Denver Instrument, Arvada. USA) to assess the quantity of sunflower oil that was absorbed by the burger, which was 7·5 g.

### Analysis of amino acid profiles

The protein content and source of each product were derived from the labels on the purchased packages. Furthermore, the contents of other macro- and, if applicable, micronutrients were derived from the front package labels as well. Although an extensive analysis is outside the scope of the present paper, their contents as derived from the front package labels of the selected products around the time of sampling are provided in online Supplementary Dataset 1. Amino acid profiles were analysed externally at Eurofins (Rotterdam, The Netherlands) following homogenisation. The total content of the AA: alanine, arginine, aspartic acid, glutamic acid, glycine, histidine, hydroxyproline, isoleucine, leucine, lysine, methionine, ornithine, phenylalanine, proline, serine, threonine, tyrosine and valine were analysed according to the ISO standard 13903:2005 (EC 152/2009). In short, samples were hydrolysed in aqueous hydrochloric acid to break peptide bonds. Subsequently, the pH of the samples was adjusted, and the samples were brought to volume with a loading buffer and filtered. The AA were then separated in an AA analyser. Detection of the AA was performed using post column derivatisation with ninhydrin reagent and 440 and 570 nm. A 1-point calibration was used for the quantification. In every run, pet food was ran as an in house standard for quality assurance. As tryptophan is decomposed during acid hydrolysis, tryptophan was analysed separately in each sample using alkaline hydrolysis, quantified by HPLC, according to EC 152/2009. Since the protein contents of the analysed analogues were derived from the front-package labels, agreement between the analysed total AA content and protein content was assessed using the Pearson correlation coefficient.

## Results

### Protein and total essential amino acid content

The protein contents of the analysed products as derived from their front package label are presented in Table 1 and visually presented in online Supplementary Figures 1–3. The average protein contents of the analysed meat and lunch meats analogues were 14·7 (sd 5·1) g/100 g and 9·2 (sd 4·4) g/100 g, respectively. The protein contents of the milk and yoghurt analogues averaged 2·5 (sd 1·3) g/100 g. The average protein content of the two cheese analogues was 6·7 (sd 2·3) g/100 g. The AA profiles of all analysed analogues and their animal-based counterparts are provided in online Supplementary Dataset 2. The correlation between the analysed total AA contents and the protein content as derived from the product labels was high (Pearson correlation coefficient 0·959 (0·92, 0·98), online Supplementary Figure 4). However, it should be noted that five of the analogues showed a relatively large difference (≥ 2·5 g) between the reported protein contents and analysed total AA content of +3·2, –6·3, –4·8, –6·3 and –10·6 g (Protein – Total AA; *Plant-Based Burger 2, Plant-Based Minced Meat 3, Seitan Sausage, Plant-Based Minced Meat 4, Plant-Based Bacon 3, respectively*). The total EAA content of the analysed meat, meats and milk and yoghurt analogues averaged 422 (sd 54), 430 (sd 48) and 414 (sd 72) mg/g protein, respectively. This averaged 302 (sd 106) mg/g protein for the two cheese analogues (online Supplementary Figures 5–7). The EAA profiles of each individual analogue are discussed in more detail in the subsequent paragraphs.

### Essential amino acid profiles

#### Meat analogues

**Figure 2.**
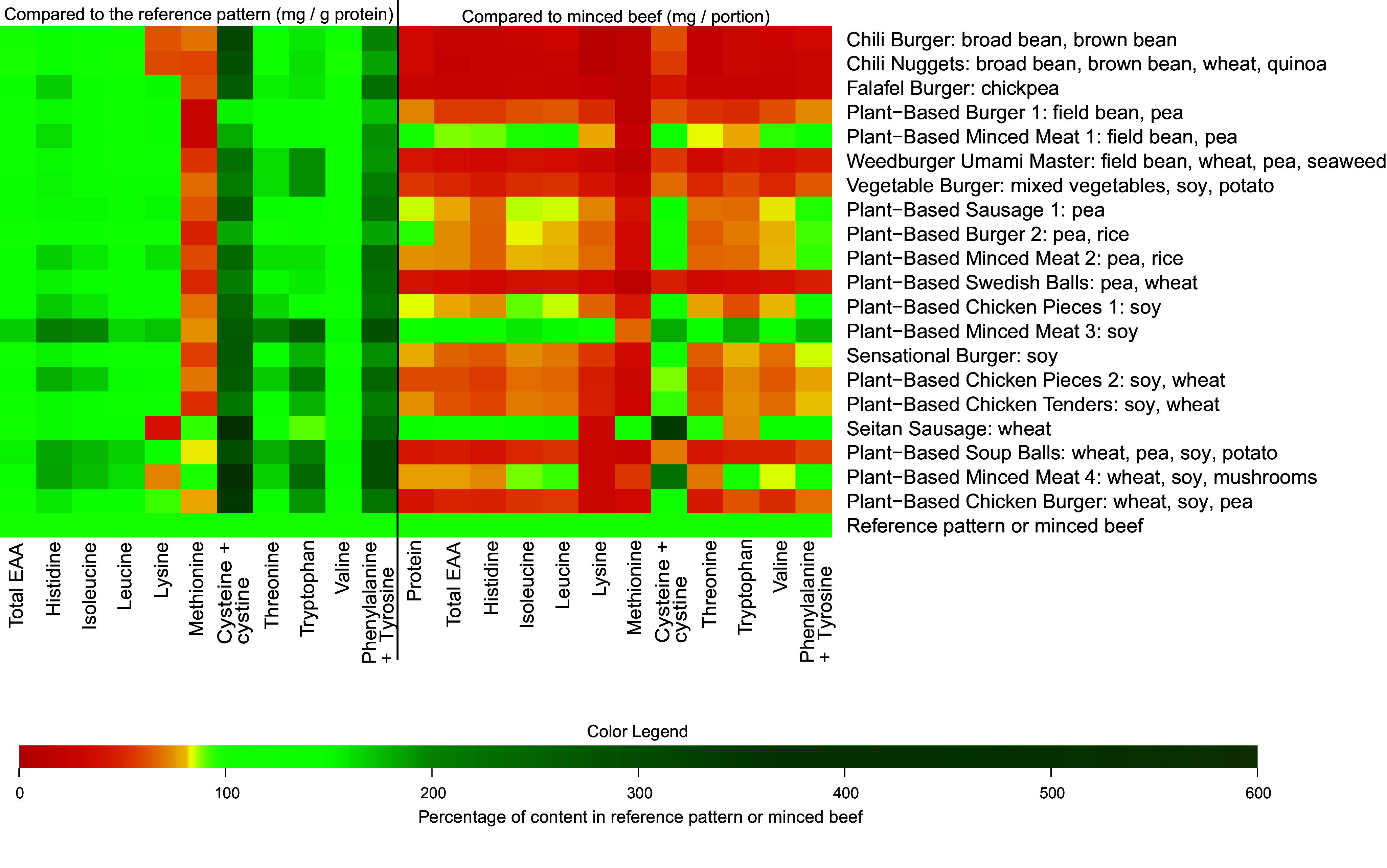
The essential amino acid profile of the analysed plant-based meat analogues in comparison with the FAO amino acid reference pattern (left) and to a portion of minced beef (right). A portion is considered 100 g for minced beef, and for the meat analogues portion sizes were calculated separately based on the package sizes, and considering usual meat portion sizes for adults. EAA, essential amino acids.

**Figure 3.**
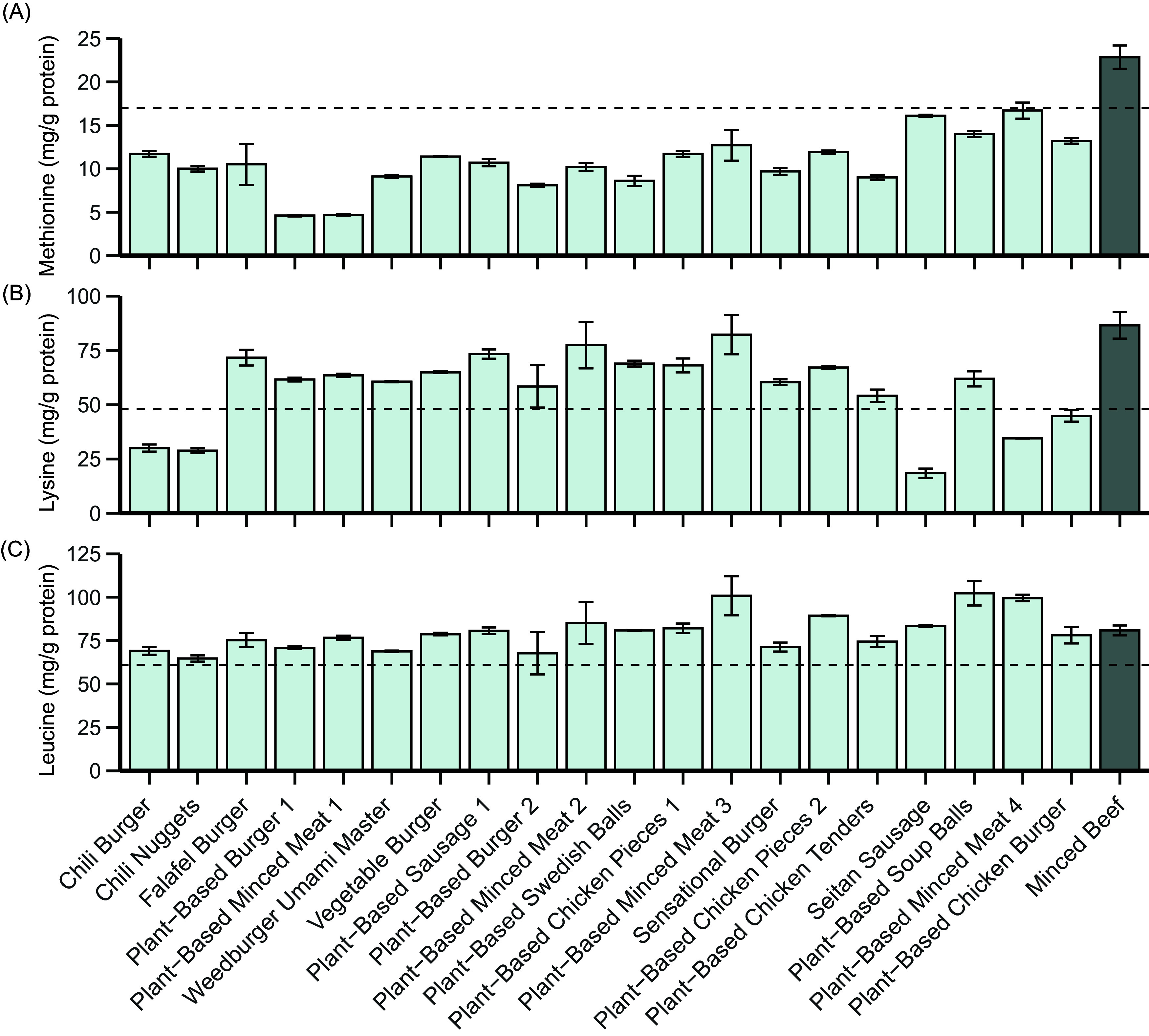
The methionine (a), lysine (b) and leucine (c) contents (mg/g protein) of the analysed meat analogues and minced beef. The dotted lines represent the reference value according to the FAO for the respective amino acid: Methionine, 17 mg/g protein; lysine, 48 mg/g protein and leucine, 61 mg/g protein. Bars represent the mean with standard deviation of the duplicate analyses performed on the sample.

Figure 2 shows how the EAA profile, additionally including the conditionally EAA cysteine + cystine and tyrosine, of each analysed meat analogue separately compares to the FAO reference pattern when expressed as mg/g protein (left) and how the quantities of these AA in a portion of each meat analogue compare to those in a portion (i.e. 100 g) of minced beef (right). Figure 3 further shows the methionine (a), lysine (b) and leucine (c) contents of each meat analogue in more detail.

Compared with the reference pattern, all meat analogues exhibited lower quantities of methionine, ranging from a 2 % lower quantity in *Plant-Based Minced Meat 4* to a 73 % lower quantity in *Plant-Based Burger 1* (Figure 3(a)). On the other hand, cysteine and its derivative cystine were present in substantially higher quantities in all meat analogues when compared with the reference pattern. Other EAA that were found in lower quantities than in the reference pattern were lysine (*n* 5) and tryptophan (*n* 1). *The Chili Burger, Chili Nuggets, Seitan Sausage, Plant-Based Minced Meat* and *Plant-Based Chicken Burger* contained lysine in quantities that were 38 %, 40 %, 62 %, 28 % and 7 % lower than the reference pattern, respectively (Figure 3(b)). The *Seitan Sausage* further contained 9·3 % lower levels of tryptophan when compared with the reference pattern (Figure 2). Leucine was adequate in all analogues when compared with the reference pattern (Figure 3(c)).

Notably, the meat analogues that incorporated the same protein sources did not necessarily reflect similar EAA profiles (Figure 2). To illustrate, when expressed as mg/g protein, the leucine contents of the meat analogues that contained soya as their only protein source were 82 (sd 3) mg/g protein (*Plant-based chicken pieces 1*), 101 (sd 11) mg/g protein (*Plant-based minced meat 3*) and 71 (sd 3) mg/g protein (*Sensational burger*). On the other hand, the two meat analogues that both incorporated field bean and pea as their only protein sources did show comparable EAA patterns, with leucine showing the largest difference between the two products (*Plant-Based Burger* 1 71 (sd 0·8) mg/g protein *v. Plant-Based Minced Meat* 1 77 (sd 1) mg/g protein).

When compared with a portion of minced beef (100 g; Figure 2), all but two meat analogues exhibited lower quantities of total EAA per portion (*Chili Burger*, –74 %; *Chili Nuggets*, –76 %; *Falafel Burger*, –74 %; *Plant–Based Burger 1*, –24 %; *Plant-Based Minced Meat 1*, –18 %; *Weedburger Umami Master*, –63 %; *Vegetable Burger*, –48 %; *Plant-Based Sausage 1*, –19 %; *Plant-Based Burger 2*, –43 %; *Plant-Based Minced Meat 2*, –24 %; *Plant-Based Swedish Balls*, –62 %; *Plant-Based Chicken Pieces 1*, –38 %; *Sensational Burger*, –32 %; *Plant-Based Chicken Pieces 2*, –19 %; *Plant-Based Chicken Tenders*, –36 %; *Plant-Based Minced Meat 4*, –43 %; *Plant-Based Soup Balls,* –53 % and *Plant-Based Chicken Burger*, –49 %). Furthermore, eleven (55 %) of the analysed meat analogues contained all individual EAA in lower quantities when compared with a portion of minced beef. On the other hand, a portion of *Plant-Based Minced Meat 3* contained greater quantities of all EAA, with the exception of methionine which was 33 % lower. All but one (*Seitan Sausage*) of the analogues exhibited lower quantities of methionine than a portion of minced beef.

**Figure 4.**
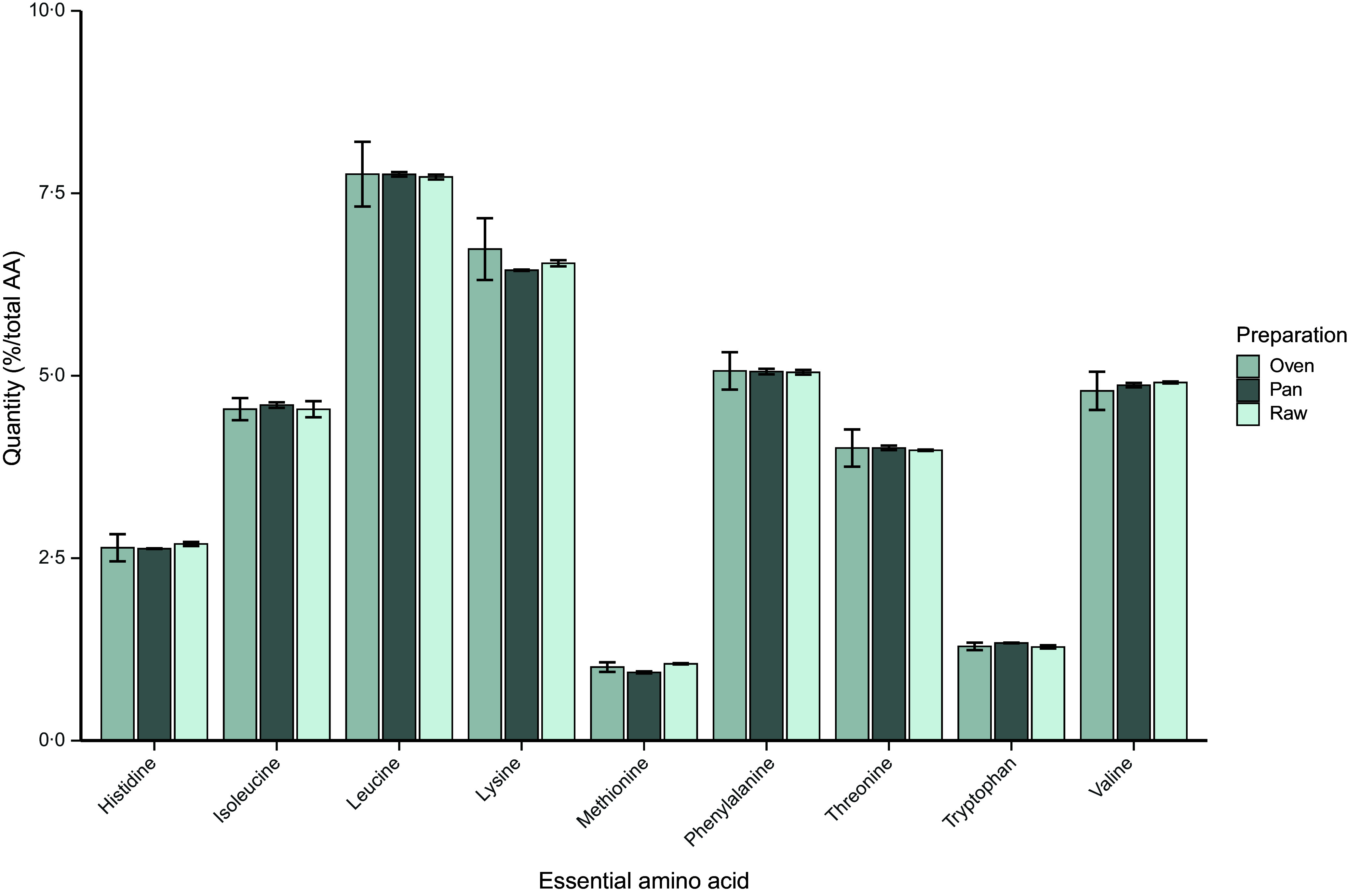
The essential amino acid profiles of the Sensational Burger when untreated and following heat processing in the oven or frying pan. To allow for direct comparisons, amino acid contents are expressed as a percentage of the sum of all amino acids in each prepared product. Bars represent the mean with standard deviation of the duplicate analyses performed on the sample.

Figure 4 shows the explorative descriptive comparison between the EAA profiles of the soya-based *Sensational Burger* following three different preparation methods. As the total protein content of the end product may change following heat treatment, we expressed EAA contents as percentage of the sum of all AA to allow for direct comparisons between the untreated, oven treated and baked analogue. The EAA composition of the untreated analogue and that of the analogues following the two different heat treatments were similar.

#### Lunch meats and cheese analogues

**Figure 5.**
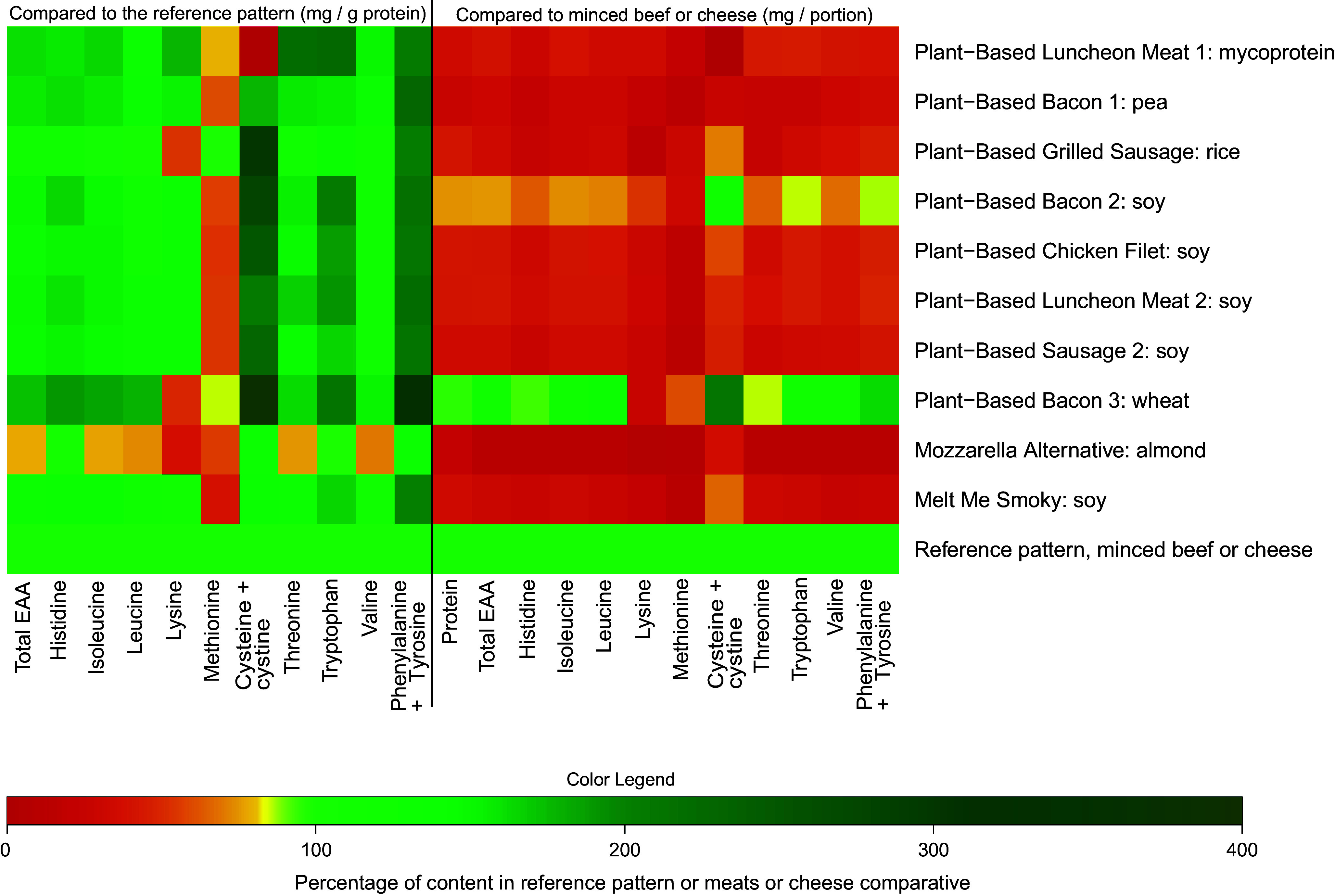
The essential amino acid profile of the analysed plant-based lunch meats- and cheese analogues in comparison to the FAO reference pattern (left) and to a bread topping portion of minced beef or cheese (right). A bread topping portion is considered 20 grams for the analogues as well as their animal-based counterparts. EAA, essential amino acids.

**Figure 6.**
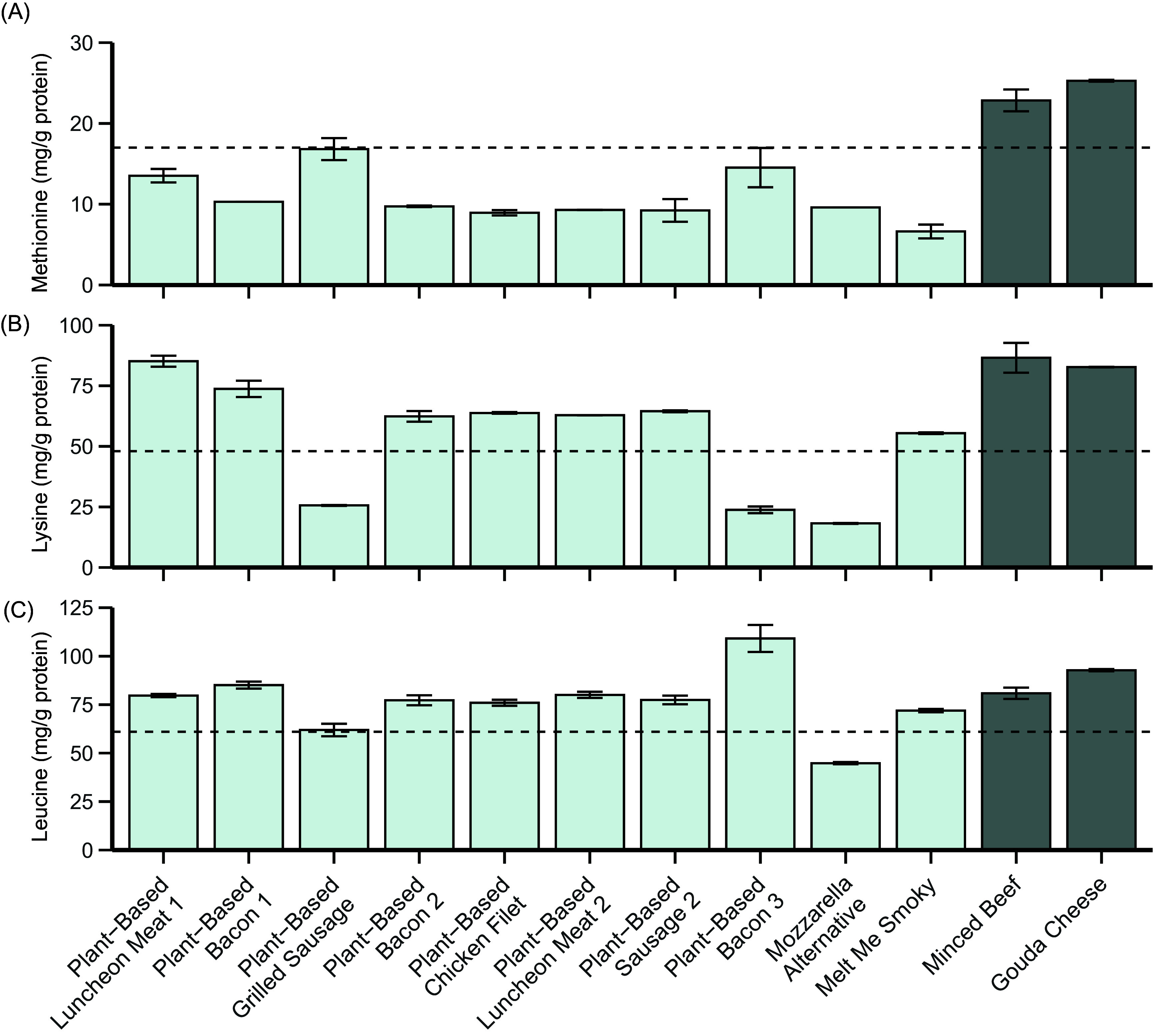
The methionine (a), lysine (b) and leucine (c) contents (mg/g protein) of the analysed lunch meats and cheese analogues, minced beef and bovine cheese. The dotted lines represent the reference value according to the FAO for the respective amino acid: Methionine, 17 mg/g protein; lysine, 48 mg/g protein; leucine, 61 mg/g protein. Bars represent the mean with standard deviation of the duplicate analyses performed on the sample.

The comparisons of the (conditional-)EAA contents of the lunch meats and cheese analogues to the reference pattern (mg/protein; left) and to a bread topping portion (i.e. 20 g) of their animal-based counterpart (right) are presented in Figure 5. The methionine (a), lysine (b) and leucine (c) contents of each lunch meat and cheese analogue are additionally presented in more detail in Figure 6.

When compared with the reference pattern, all lunch meats and cheese analogues exhibited lower quantities of methionine (*Plant-Based Luncheon meat 1*, –20 %; *Plant-Based Bacon 1*, –39 %; *Plant-Based Grilled Sausage*, –1 %; *Plant-Based Bacon 2*, –43 %; *Plant-Based Chicken filet*, –47 %; *Plant-Based Luncheon Meat 2*, –45 %; *Plant-Based Sausage 2*, –46 %; *Plant-Based Bacon 3*, –15 %; *Mozzarella Alternative*, –44 % and *Melt Me Smoky*, –61 %; Figure 6(a)). Additionally, three analogues exhibited lower quantities of lysine when compared with the reference pattern (*Plant-Based Grilled Sausage*, –47 %; *Plant-Based Bacon* 3, –50 % and *Mozzarella Alternative*, –62 %; Figure 6(b)). Furthermore, the almond-based *Mozzarella Alternative* additionally exhibited lower quantities of isoleucine (–23 %), leucine (–27 %), threonine (–25 %) and valine (–30 %). The other analogues all contained adequate quantities of leucine (Figure 6(c)). *Plant-Based Luncheon Meat 1* did not contain any cysteine + cystine, while all other analogues exhibited relatively higher values, ranging from 30 % higher quantities in *Mozzarella Alternative* to 292 % higher quantities in *Plant-Based Bacon 3* (Figure 5).

Compared with a portion (20 g) of their animal-based counterpart, protein content per portion was lower in all lunch meats and cheese analogues, which is reflected in their more unfavourable EAA compositions (Figure 5). Two analogues contained one or more (conditional-)EAA in higher quantities than a portion of minced beef. *Plant-Based Bacon 2* showed 17 % higher quantities of cystine + cysteine when compared with minced beef. *Plant-Based Bacon 3* exhibited higher quantities of isoleucine (+18 %), leucine (+28 %), cystine + cysteine (+111 %), tryptophan (+13 %), valine (+11 %) and phenylalanine + tyrosine (+63 %). All other analogues exhibited all (conditional-)EAA in lower quantities compared with a portion of their animal-based counterpart, ranging from the absence of cystine + cysteine in *Plant-Based Luncheon Meat 1* to a 7 % lower quantity of histidine in *Plant-Based Bacon 3*. Notably, the four lunch meats analogues that contained soya as their only protein source (*Plant-Based Bacon 2, Plant-Based Chicken Filet, Plant-Based Luncheon Meat 2 and Plant-Based Sausage 2*) all exhibited similar EAA profiles when expressed as mg/g protein, but not per portion due to differences in total protein content (Table 1; online Supplementary Dataset 1).

#### Milk and yoghurt analogues

**Figure 7.**
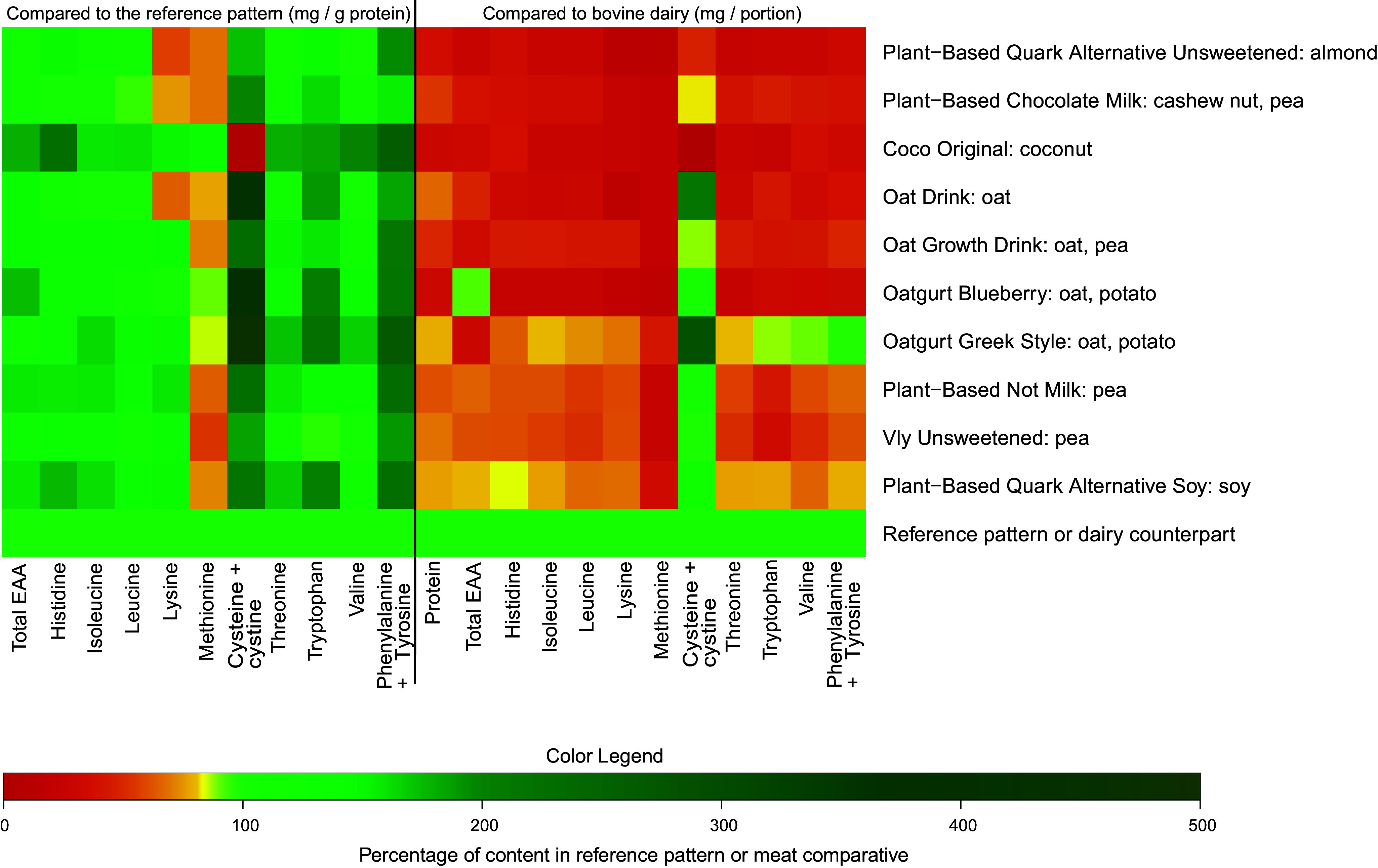
The essential amino acid profile of the analysed plant-based milk and yoghurt analogues in comparison with the FAO reference pattern (left) and to a portion of their animal-based comparative (right). A portion is considered 150 grams. Note: Considering varying protein contents in different dairy products, when comparing the analogues to their animal-based counterpart, the EAA contents of Plant-Based Quark Alternative Unsweetened and Plant-Based Quark Alternative Soya were compared to that in a portion of bovine semi-skimmed quark (11·3 g protein/portion). The EAA content of Plant-Based Chocolate Milk was compared to that in a portion of bovine full-fat bovine chocolate milk (5·0 g protein/portion). The EAA contents of Coco Original, Oatgurt Blueberry and Oatgurt Greek style were compared with that of semi-skimmed bovine yoghurt (6·3 g protein/portion). All other analogues were compared with a portion of bovine semi-skimmed milk (5·4 g protein/portion). EAA, essential amino acids.

**Figure 8.**
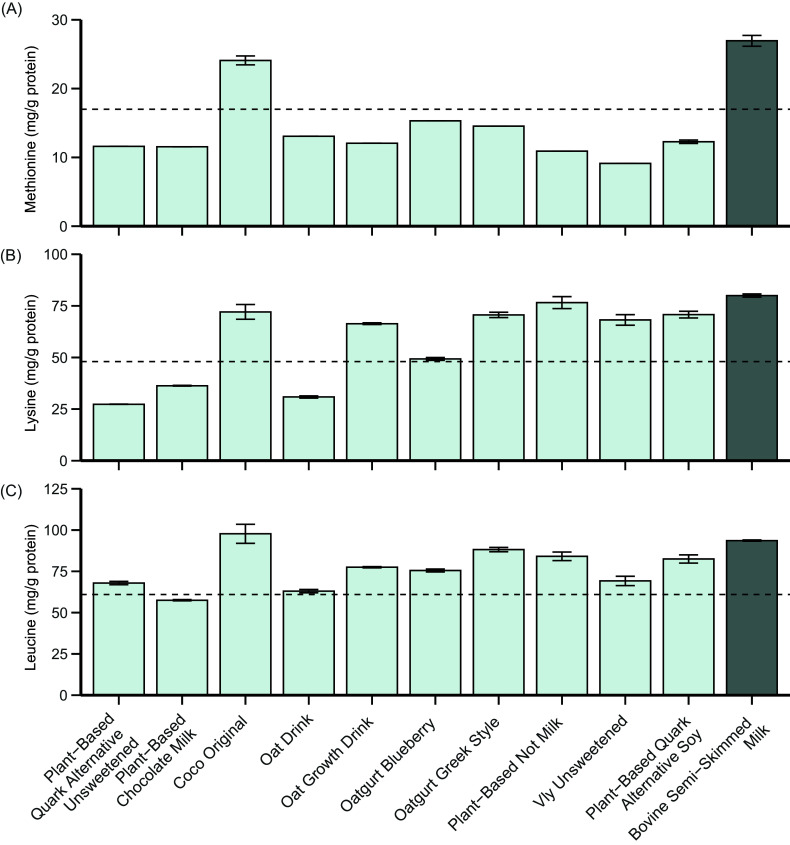
The methionine (a), lysine (b) and leucine (c) contents (mg/g protein) of the analysed milk and yoghurt analogues and semi-skimmed bovine milk. The dotted lines represent the reference value according to the FAO for the respective amino acid: Methionine, 17 mg/g protein; lysine, 48 mg/g protein and leucine, 61 mg/g protein. Bars represent the mean with standard deviation of the duplicate analyses performed on the sample.

Figure 7 displays the (conditional-)EAA content of the milk and yoghurt analogues compared with the reference pattern (mg/g protein; left) and to a portion of their animal-based counterpart (right). The methionine (a), lysine (b) and leucine (c) contents are again separately presented in more detail in Figure 8. Compared with the reference pattern, leucine (*n* 1), methionine (*n* 9), lysine (*n* 3), cystine + cysteine (*n* 1) and tryptophan (*n* 1) contents were lower in several of the analysed milk and yoghurt analogues (Figure 7). *Plant-Based Chocolate Milk* and*Plant-Based Quark Alternative Unsweetened* and*Oat Drink* contained lesser quantities of both methionine (32 %, 32 % and 23 %, respectively) and lysine (24 %, 43 % and 36 %, respectively; Figure 8(a) and (b)). The products that only exhibited lower quantities of methionine than the reference pattern were *Oat Growth Drink* (–29 %), *Oatgurt Blueberry* (–3 %), *Oatgurt Greek Style* (–47 %), *Plant-Based Not Milk* (–60 %) and *Plant-Based Quark Alternative Soya* (–48 %). *Vly Unsweetened* contained lower quantities of both methionine (–46 %) and tryptophan (–4 %). Although the product *Coco Original* exhibited quantities of methionine and lysine that were higher than those in the reference pattern, it did not contain cysteine or its derivative cystine. Leucine contents were adequate for all analogues, with the exception of *Plant-Based Chocolate Milk*, which exhibited a 6 % lower quantity of leucine than the reference pattern (Figure 8(c)). The analogues exhibited the other EAA in similar or higher quantities compared with the reference pattern, ranging from a 2 % higher quantity of isoleucine in *Oat Drink* to a 380 % higher quantity of cystine + cysteine in *Oatgurt Greek Style* (Figure 7).

When compared with a portion (i.e. 150 g) of their animal-based counterpart (Figure 7), six (60 %) milk and yoghurt analogues (*Plant-Based Quark Alternative Unsweetened, Plant-Based Chocolate Milk, Coco Original, Oat Growth Drink, Oatgurt Blueberry, Vly Unsweetened*) exhibited lower quantities of all EAA, ranging from the absence of cystine + cysteine in *Coco Original* to a 1 % lower quantity of cystine + cysteine in *Oatgurt Blueberry*. The remaining four milk and yoghurt analogues exhibited higher quantities of cystine + cysteine than their dairy counterpart, but contained lower quantities of all other EAA.

## Discussion

The accelerated production of plant-based meat and dairy analogues in high-income countries, driven by the protein transition, demands to better understand their nutrient composition. In this study we assessed the protein contents and AA profiles of 40 different fully plant-based meat and dairy analogues. Most analogues displayed lower quantities of protein than their animal-based counterparts, which was also reflected in lower EAA contents. While many EAA and the sum of the total EAA contents reached the estimated human EAA requirements established by the FAO^(9)^ in most of the analogues, none of the analogues displayed a complete EAA profile. Insufficiencies of methionine and lysine contents were most frequently observed.

With the exception of one analogue, all analogues exhibited relatively low quantities of methionine compared to the reference pattern or their animal-based counterpart. More specifically, low quantities of methionine and lysine were observed in 39 and 11 of the 40 analogues when compared to the reference pattern, respectively. Our findings are in line with previous studies, which have reported low quantities of methionine and/or lysine in various plant-based protein isolates^(11,13)^, meat- and dairy analogues^(4,8,21–23)^, plant-centered hospital meals^(24)^, as well as low dietary intakes in individuals consuming plant-based diets^(25,26)^. Adequate consumption of methionine is, because of its sulfur atom, required for distinctive functions beyond its human requirements for protein synthesis. Methionine is, for example, additionally involved in the synthesis of cysteine and methyl nutrients, such as creatine, via transsulfuration and transmethylation^(27)^. With regards to lysine, its main role lies in its participation in the synthesis of new tissue proteins, but lysine carries other important functions as well, for example, participating in fatty acid metabolism and calcium absorption^(28,29)^. Considering that methionine and lysine are typically the limiting EAA in plant-based products and plant-centered diets, the low quantities observed here are not surprising. Nevertheless, the opportunity exists for the industry to optimise the nutritional contents of meat and dairy analogues, particularly focusing on EAA like methionine and lysine, during the production process to better meet consumers’ nutritional needs. This may be achieved by fortification with the deficient free amino acid(s), increasing protein content and/or by applying a protein blend of both plant- and animal-based proteins

Notably, we observed low quantities of methionine and lysine in meat and dairy analogues that were based on protein sources that in isolated form do not necessarily contain low quantities of these AA. For example, we observed that the meat analogues that incorporated both soya and wheat still exhibited relatively low quantities of lysine or methionine, while being reportedly complementary in their AA profiles^(11,13)^. This is in line with a previous study^(21)^, that observed incomplete EAA patterns in meat analogues that incorporated both legumes and cereals as a protein source. Furthermore, we observed that analogues incorporating proteins from comparable sources (e.g. soya) did not consistently exhibit similar AA profiles. Although surprising, this may be explained by several factors. First, the protein sources may be incorporated in unfavorable quantities or combined in suboptimal proportions that fail to meet EAA requirements. Second, the protein sources may have been incorporated in other forms than as isolated proteins, e.g. as flour, providing much less protein per g product. Third, frequently applied processing techniques may alter the AA composition of the end product^(16,30)^. The latter being of particular relevance for the meat analogues, which frequently undergo several intensive processing steps during the production process, such as extrusion cooking and shear structuring^(16,30)^. Due to the high temperatures used during these processes, some of the EAA may be degraded. Lysine, for example, may be degraded as a consequence of Maillard reactions^(16,30)^.

With the exception of methionine and lysine, adequate amounts of all other EAA were present in most analogues. The consumption of the branched chain AA leucine, isoleucine and valine has previously been observed to be low in individuals following a plant-based diet as well^(25,26)^. Aaslyng *et al.* ^(25)^ observed that 30 % of their vegan participants did not consume the required quantity of leucine on all three days that dietary intake was assessed. Schmidt *et al.* ^(26)^ observed ∼30 % lower intakes of leucine, isoleucine and valine in vegans when compared to meat eaters. We observed adequate quantities of these AA in all but two of the analysed analogues when compared with human requirements. Leucine in particular is known for its anabolic potency due to its ability to directly activate cellular pathways that stimulate muscle protein synthesis and is therefore considered an important prerequisite for the ability of a protein source to stimulate post-prandial muscle protein synthesis^(31–33)^. However, only two (*Plant-Based Minced Meat 3; Seitan Sausage*) analogues assessed here exhibited per portion the quantity of leucine that is often proposed as necessary to ‘trigger’ protein synthesis, namely ∼2–3 g. Nevertheless, this leucine ‘trigger’ hypothesis, especially in the context of whole foods, has been challenged^(34)^. Also, once triggered, the synthesis of new bodily proteins still requires all EAA to serve as precursors, and inadequacy of one or more EAA may still compromise the post-prandial muscle protein synthetic response^(11,12)^.

A healthy diet that is rich in various (plant-based) protein sources and additionally includes meat and dairy analogues is unlikely to result in protein or specific AA deficiencies in healthy adults. However, in vulnerable individuals, such as older adults, clinically compromised individuals, or children, substituting meat and dairy by their plant-based analogues may be a reason for concern, as meeting their increased protein requirements is already challenging on a diet that does include meat and dairy^(35,36)^. Our findings are additionally relevant for individuals who exclude all animal-based food products from their diet, as they are recommended to increase their dietary protein intake as well, to compensate for the lower protein digestibility of plant-based foods and, therefore, need to carefully choose their plant-based meat and dairy substitutions^(37)^. As such, we recommend several strategies to overcome the lower protein quality that results from the lower content of one or more specific EAA in these products. First, we recommend to consume meals that include a variety of food products, that combined in a meal are no longer deficient in specific amino acids. For example, wheat products, such as bread, pasta and rice are rich in methionine. By combining a meat analogue with rice, or a dairy analogue with bread, methionine contents of the respective meal may become more balanced. A helpful tool in composing meals that exhibit complete AA profiles is The Meal Protein Quality Score^(24)^. Second, although potentially not feasible for individuals with a reduced appetite^(35)^, increasing the total protein content of the respective meal by including more high-protein foods, or increasing the proportion of the high-protein foods within the meal, may result in a meal with a more complete and balanced EAA profile. Third, it should be noted that our findings were observed in fully plant-based analogues. The moderate addition of some animal-based food sources to the respective meal, or within the respective analogue, may improve the EAA composition and, as such, prevent any specific amino acid deficiencies of the product or the respective meal.

We acknowledge that the choice for substituting (more) meat or dairy by their plant-based analogues may be predominantly driven by environmental concerns rather than health factors, considering the relatively higher reported environmental impact of animal-based foods^(38,39)^. However, understanding their (protein) nutritional quality is irrespectively essential for a healthy transition towards more sustainable diets. The findings presented here may further substantiate the more sustainable dietary guidelines that are envisaged in the near future.

Several limitations of the present study should be addressed. First, although we provided a supplementary dataset presenting nutrient constituents of the analogues other than protein and AA, these components were not extensively assessed. The AA composition of meat and dairy analogues is a pivotal determinant for their nutritional quality. However, other nutrients should be considered as well to provide an overview of the overall nutritional quality of meat and dairy analogues. This was outside the scope of the present paper, as the macronutrient composition of meat- and dairy analogues has previously and extensively been assessed by others^(38,40)^. Second, there is a rapid (dis)appearance of meat and dairy analogues from the Dutch market. This impedes our understanding in the nutrient quality of these products. Particularly considering our finding that the ingredients are not necessarily informative of the AA profile of the analogue. The analogues assessed in this study were purchased and analysed in March 2023. As such, potentially, some of the analogues analysed in this study, may have already been removed from the Dutch market, while other analogues may have appeared on the market that potentially exhibit different AA compositions. Last, the protein contents of the analysed analogues were derived from the front-package labels. Manufacturers are required to use the nitrogen-to-protein conversion factor of 6·25 for the determination of total protein content in food products (EU regulation nr. 1169/2011). While generally adopted, this factor tends to overestimate the protein content of many plant-based foods^(41)^. Still, we observed a high correlation between total analysed AA contents and the protein contents reported on the product labels (Pearson correlation coefficient 0·959). Nevertheless, we observed relatively large differences between the reported protein content and the analysed total AA content for five of the analysed products. It is unknown whether these differences are caused by measurement errors during the determination of the AA contents. However, since the AA profiles of the respective products are generally in line with our observations in the other products, we feel that the impact on our findings is low.

This study reported the AA profiles of a broad range of meat and dairy analogues available on the Dutch market. By reporting the AA profiles individually for each analogue, the data from this study can be applied in nutrient databases, which is a major strength of our study. Furthermore, the assessment of analogues that were based on a variety of protein sources and the comparisons with human requirements and animal-based counterparts deepens our understanding regarding the protein quality of the respective products and their heterogeneity. Our findings may catalyse enhancements in the product development process that could improve the AA profile of meat and dairy analogues. Our findings may further improve consumer choices by providing insights into the AA compositions of the analogues. Future studies are encouraged to consider the protein digestibility and functionality of different meat- and dairy analogues when consumed in isolated form and as constituent of a mixed meal or complete diet.

In conclusion, meat- and dairy analogues frequently display lower protein contents than their animal-based counterparts and exhibit incomplete EAA profiles. Lysine, but in particular methionine, is the most frequently limiting AA in these products. On the other hand, adequate quantities of the other EAA were observed in most analogues. Moreover, the combination of protein sources used in the formulation of an analogue do not guarantee an adequate AA composition, as the form and quantity of the incorporated sources, ingredient interactions and more importantly, processing methods, may alter the final AA profile of the dairy or meat analogue.

## Supporting information

Domić et al. supplementary material 1 Domić et al. supplementary material

Domić et al. supplementary material 2 Domić et al. supplementary material

Domić et al. supplementary material 3 Domić et al. supplementary material

## Supplementary material

For supplementary material/s referred to in this article, please visit https://doi.org/10.1017/S000711452510408X
