# Supplementary material for: The amino acid composition of commercially available vegan meat and dairy analogues
Source: Br J Nutr. 2025 Jul 31;134(5):377–89. doi: 10.1017/S000711452510408X (PMC12580972; doi:10.1017/S000711452510408X)
Supplement: Domić et al. supplementary material 1 — Domić et al. supplementary material [file S000711452510408Xsup001.docx]

Supplementary material belonging to:

# **The amino acid composition of commercially available vegan meat and dairy analogues**

Jacintha Domić^1^, Luc JC van Loon^2^, Els Siebelink^1^, Karin J Borgonjen-van den Berg^1^, Lisette CPGM de Groot^1^, Pol Grootswagers^1^

1 Division of Human Nutrition and Health, Wageningen University, Wageningen, The Netherlands.

2 Department of Human Biology, Institute of Nutrition and Translational Research in Metabolism (NUTRIM), Maastricht University Medical Centre+, Maastricht, The Netherlands.


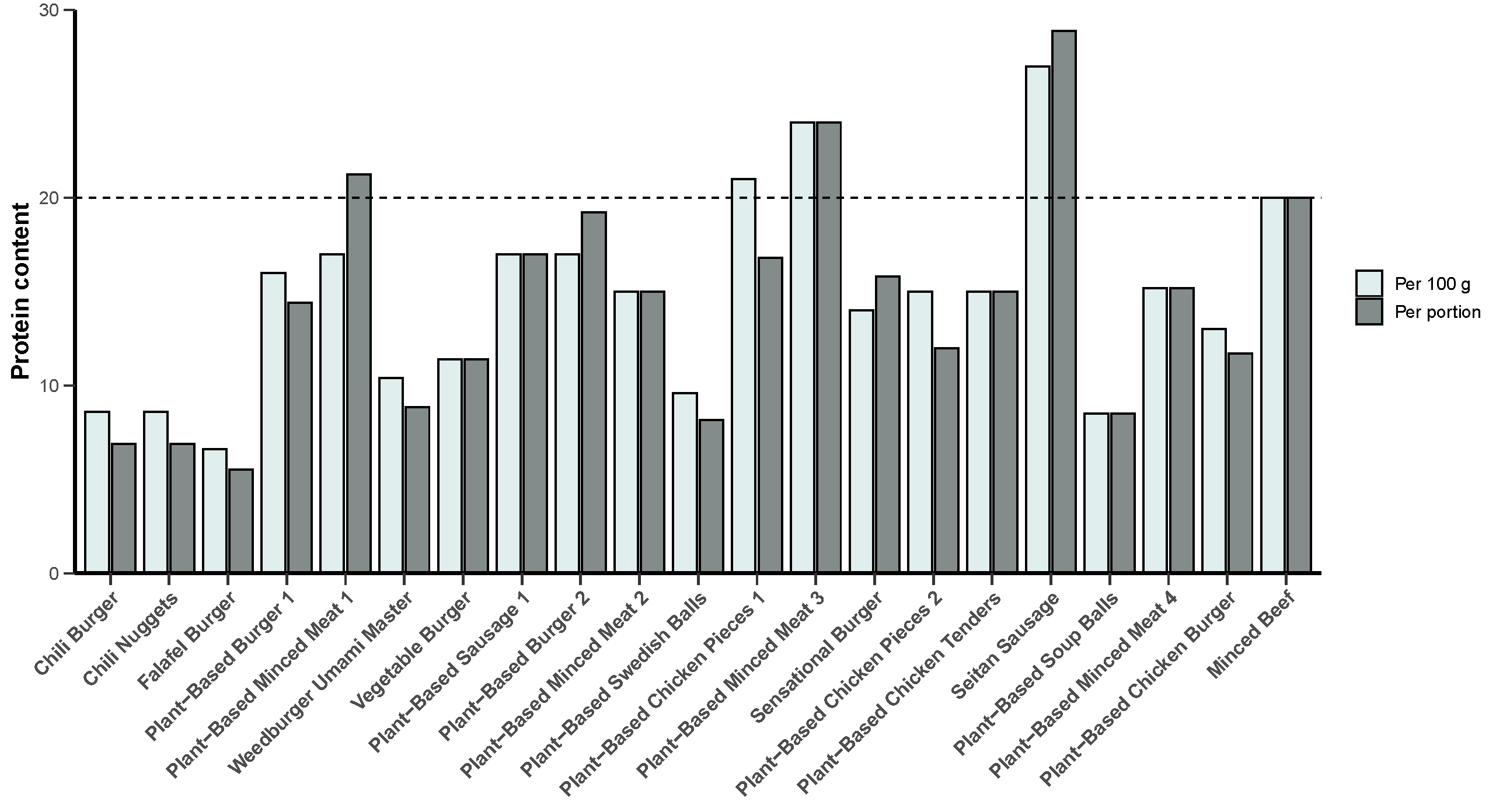
**Supplementary figure 1.** Protein content of the analyzed plant-based meat analogues and minced beef.

The dashed line represents the protein content per 100 g of minced beef.


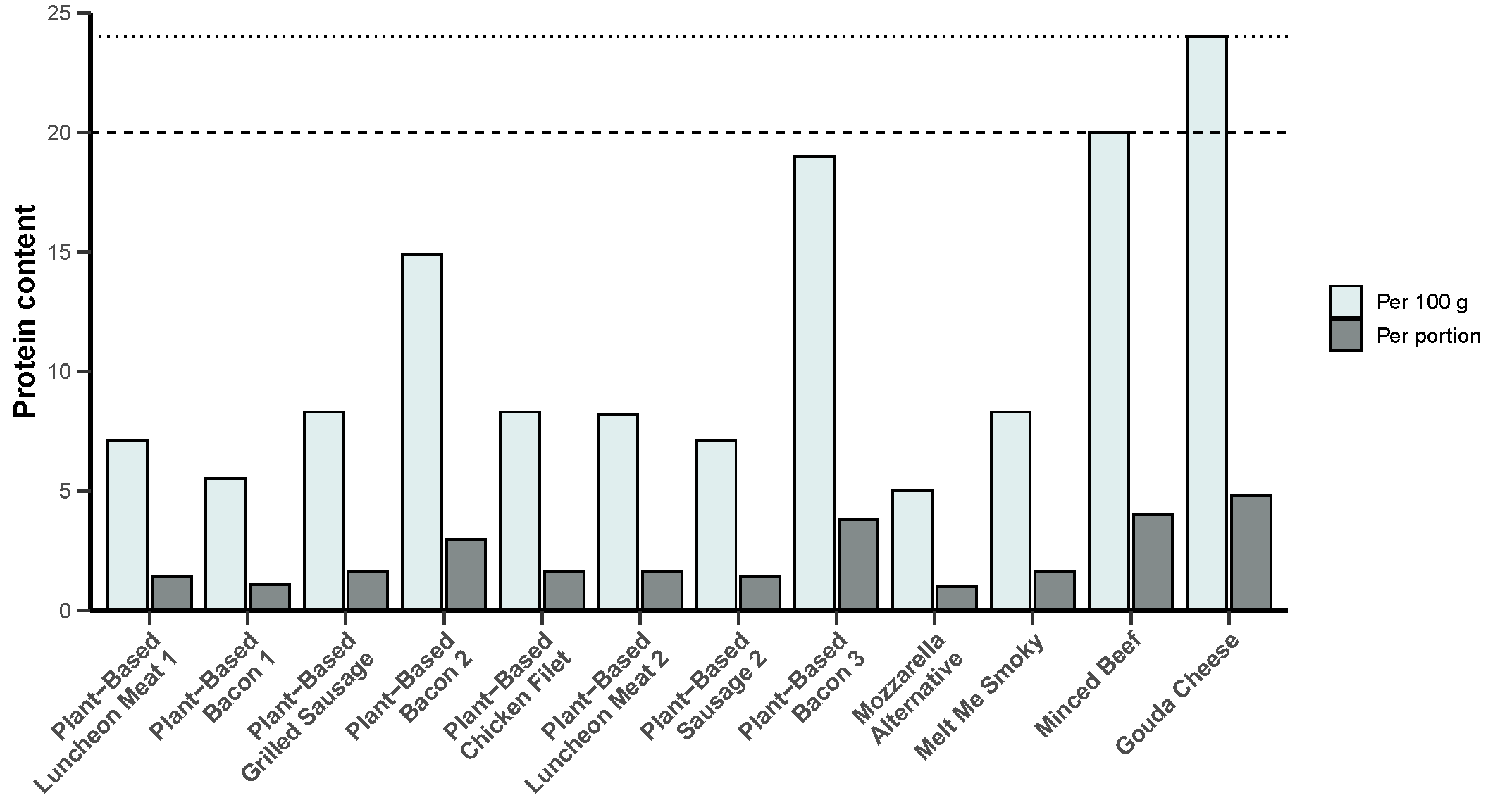


**Supplementary figure 2.** Protein content of the analyzed plant-based meats and cheese analogues and minced beef and bovine cheese.

The dashed and dotted lines represent the protein content per 100 g of minced beef and cheese, respectively.


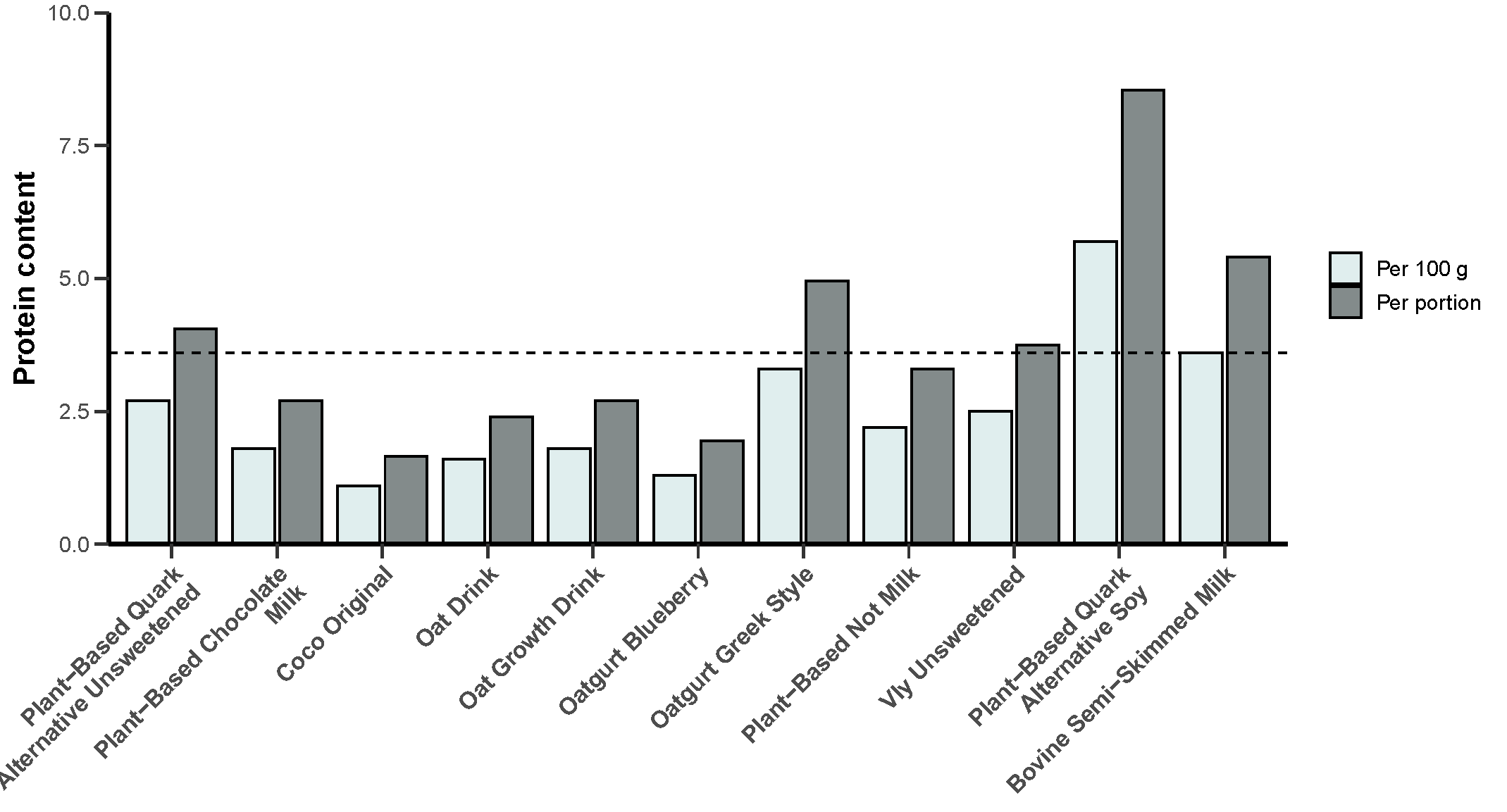


**Supplementary figure 3.** Protein content of the analyzed plant-based milk and yoghurt analogues and bovine semi-skimmed milk.

The dashed line represents the protein content per 100 g of bovine semi-skimmed milk.


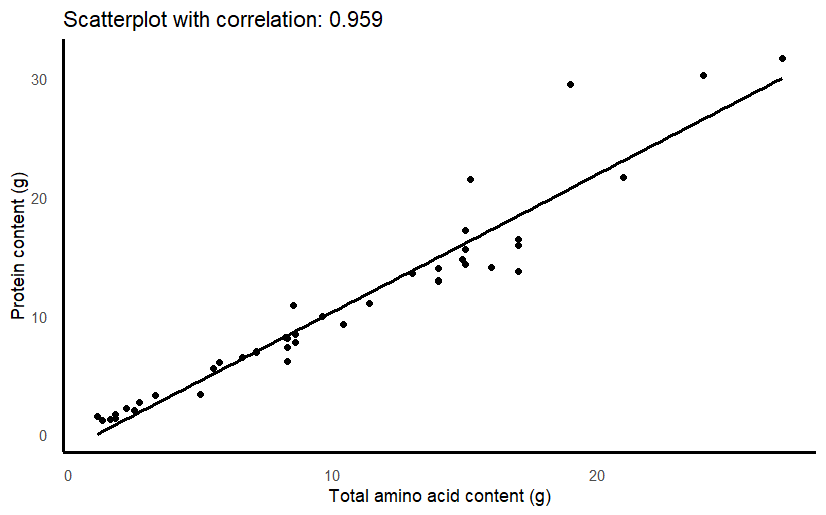


**Supplementary figure 4.** Correlation between the analyzed total amino acid content and the protein content in g/100 g product as derived from the front-package label. Pearson correlation coefficient 0.959 (0.92, 0.98).


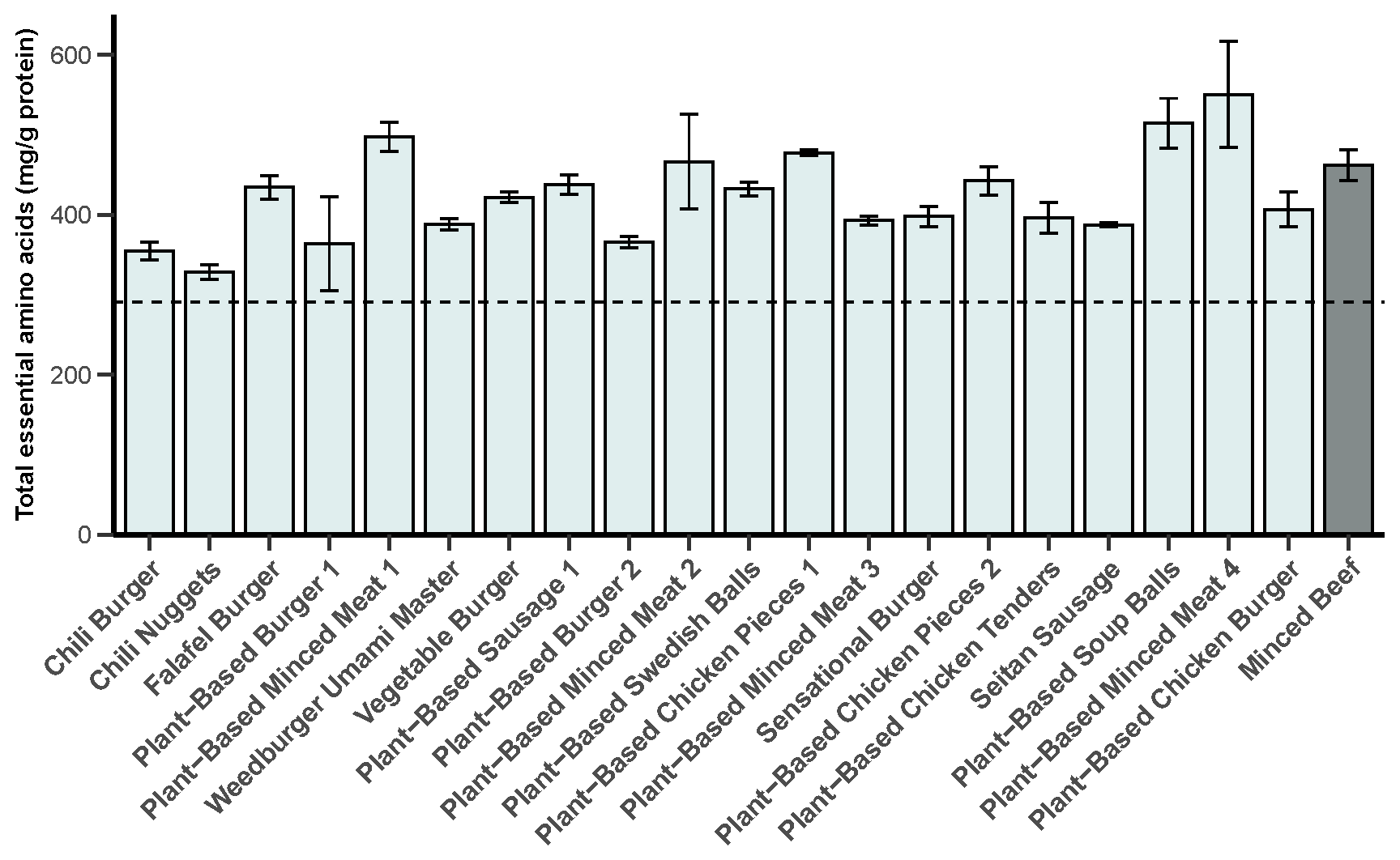


**Supplementary figure 5.** Total essential amino acid content of the analyzed plant-based meat analogues and minced beef.

The dashed line represents the total (conditional-)EAA content of the FAO reference pattern (290.6 mg/g protein).


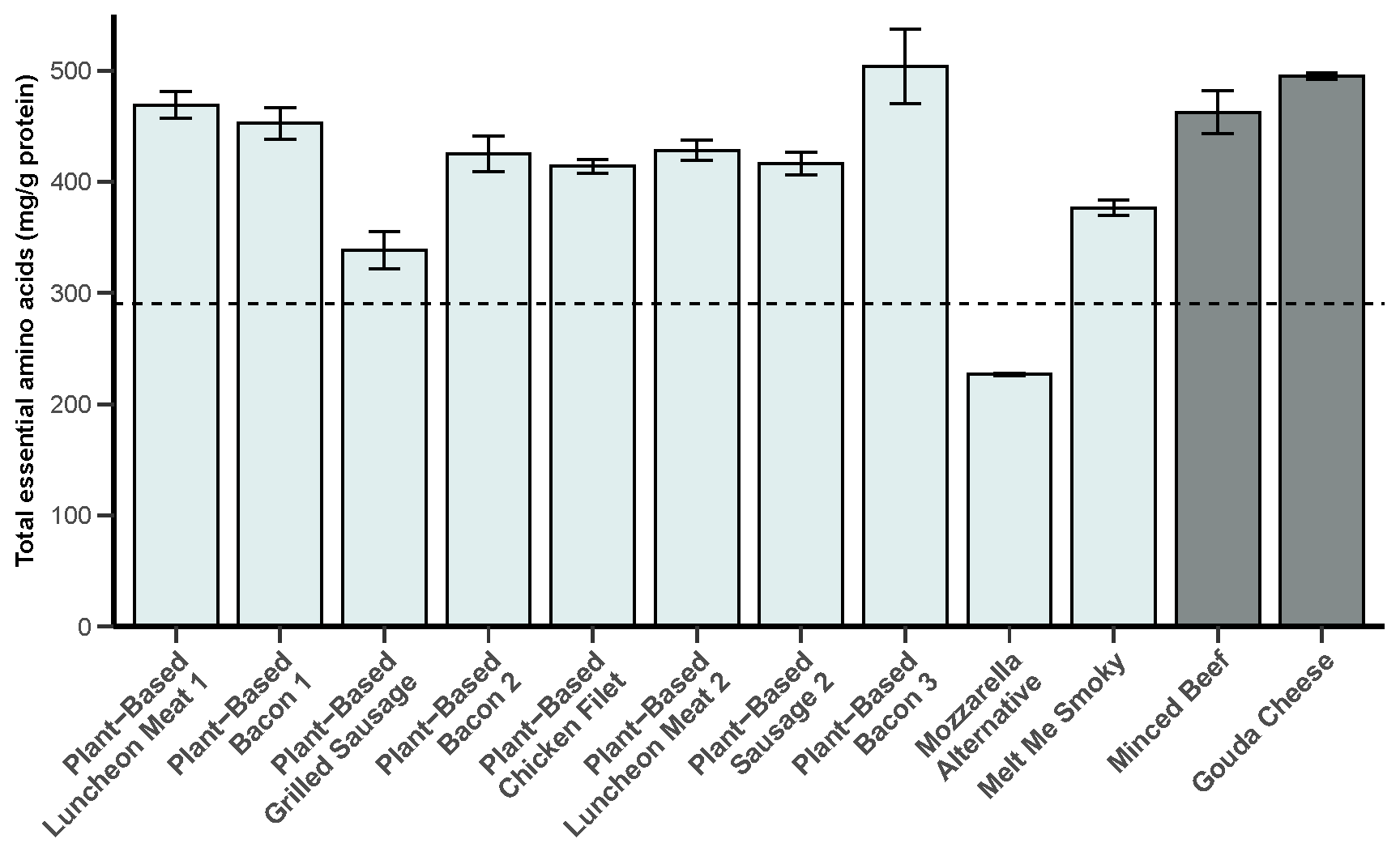


**Supplementary figure 6.** Total essential amino acid content of the analyzed plant-based meats- and cheese analogues, minced beef and bovine Gouda cheese.

The dashed line represents the total (conditional-)EAA content of the FAO reference pattern (290.6 mg/g protein).


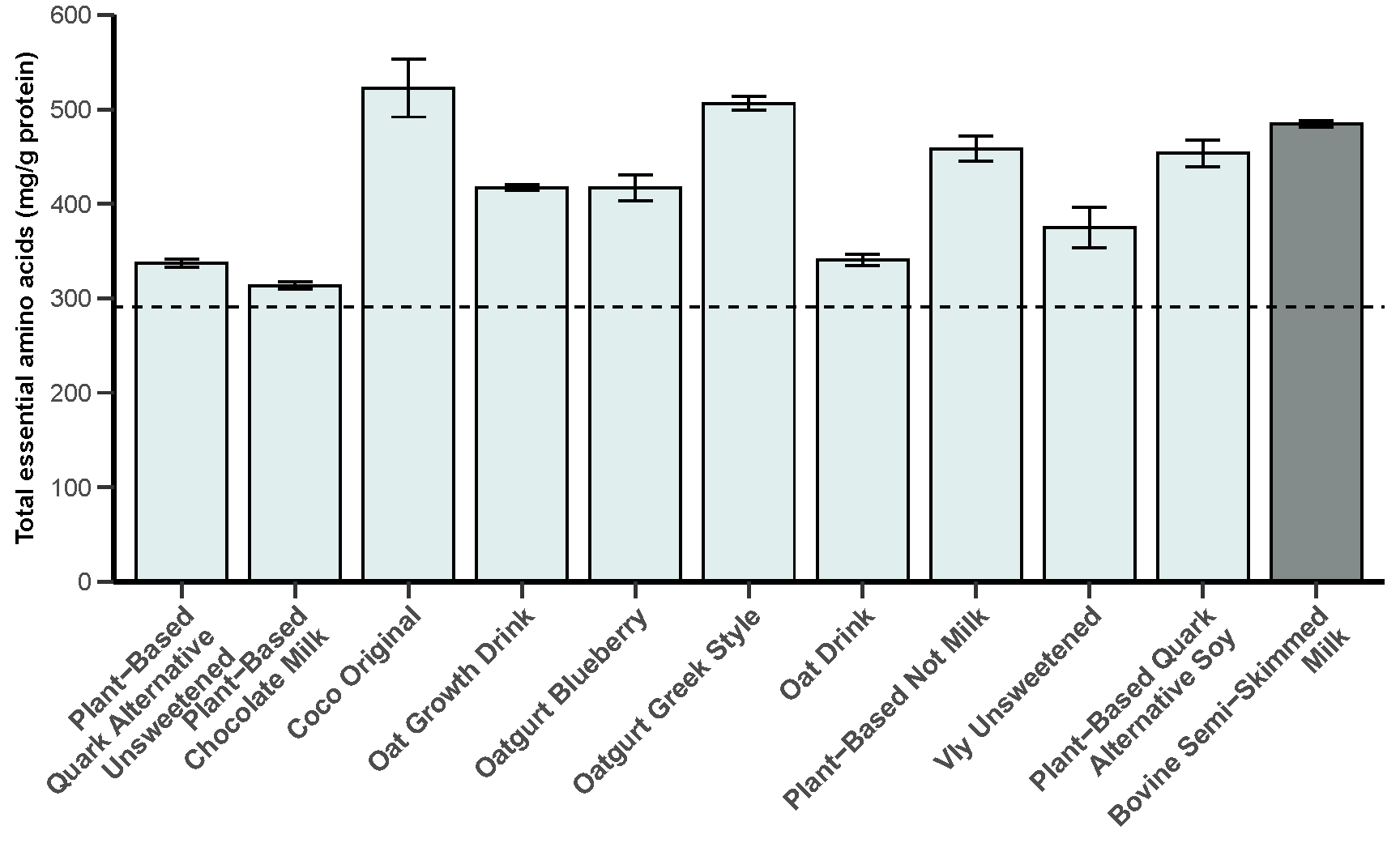


**Supplementary figure 7.** Total essential amino acid content of the analyzed plant-based milk- and yoghurt analogues, and bovine semi-skimmed milk.

The dashed line represents the total (conditional-)EAA content of the FAO reference pattern (290.6 mg/g protein).
